# Sex‐related effects in major depressive disorder: Results of the European Group for the Study of Resistant Depression

**DOI:** 10.1002/da.23165

**Published:** 2021-06-10

**Authors:** Lucie Bartova, Markus Dold, Gernot Fugger, Alexander Kautzky, Marleen M. M. Mitschek, Ana Weidenauer, Marius G. Hienert, Richard Frey, Laura Mandelli, Joseph Zohar, Julien Mendlewicz, Daniel Souery, Stuart Montgomery, Chiara Fabbri, Alessandro Serretti, Siegfried Kasper

**Affiliations:** ^1^ Department of Psychiatry and Psychotherapy Medical University of Vienna Vienna Austria; ^2^ Department of Biomedical and NeuroMotor Sciences University of Bologna Bologna Italy; ^3^ Psychiatric Division Chaim Sheba Medical Center Tel Hashomer Israel; ^4^ School of Medicine Free University of Brussels Brussels Belgium; ^5^ Psy Pluriel ‐ European Centre of Psychological Medicine Brussels Belgium; ^6^ Imperial College School of Medicine University of London London United Kingdom; ^7^ Social, Genetic and Developmental Psychiatry Centre, Institute of Psychiatry, Psychology and Neuroscience King's College London London United Kingdom; ^8^ Center for Brain Research Medical University of Vienna Vienna Austria

**Keywords:** gender, major depressive disorder, male depression, sex, treatment response

## Abstract

**Background:**

Sex‐related effects on the evolution and phenotype of major depressive disorder (MDD) were reported previously.

**Methods:**

This European multicenter cross‐sectional study compared sociodemographic, clinical, and treatment patterns between males and females in a real‐world sample of 1410 in‐ and outpatients with current MDD.

**Results:**

Male MDD patients (33.1%) were rather inpatients, suffered from moderate to high suicidality levels, received noradrenergic and specific serotonergic antidepressants (ADs) as first‐line AD treatment, generally higher mean AD daily doses, and showed a trend towards a more frequent administration of add‐on treatments. Female MDD patients (66.9%) were rather outpatients, experienced lower suicidality levels, comorbid thyroid dysfunction, migraine, asthma, and a trend towards earlier disease onset.

**Conclusions:**

The identified divergencies may contribute to the concept of male and female depressive syndromes and serve as predictors of disease severity and course, as they reflect phenomena that were repeatedly related to treatment‐resistant depression (TRD). Especially the greater necessity of inpatient treatment and more complex psychopharmacotherapy in men may reflect increased therapeutic efforts undertaken to treat suicidality and to avoid TRD. Hence, considering sex may guide the diagnostic and treatment processes towards targeting challenging clinical manifestations including comorbidities and suicidality, and prevention of TRD and chronicity.

## 1 INTRODUCTION

Relevant sex‐related effects on the evolution and phenotype of major depressive disorder (MDD) were reported previously (Parker & Brotchie, 2010; Salk et al., 2017). Hereby, MDD was shown to emerge twice as common in women as in men (Kornstein, 1997). Furthermore, clinical manifestations (Grigoriadis & Robinson, 2007), coping with depressive symptoms (Angst et al., 2002), and help‐seeking patterns (Moller‐Leimkuhler, 2002; Oliffe et al., 2019) were repeatedly shown to differ between female and male MDD patients. While MDD in women was associated with seasonal components, anxiety, affective lability, and atypical features, the concept of the so‐called male depression characterized by agitation, increased impulsivity, irritability, risk‐taking behavior, and comorbid substance misuse was introduced (Azorin et al., 2014; Moller‐Leimkuhler, 2009; Oliffe et al., 2019; Rutz et al., 1995; Winkler et al., 2004; 2005). Additionally, sex was shown to influence antidepressant (AD) treatment in terms of efficacy and pharmacokinetics (Sramek et al., 2016), though not unequivocally (De Carlo et al., 2016; Serretti et al., 2009). Possible explanations for these sex differences were studied in animal models (Ma et al., 2019) as well as in humans, whereby psychological and personality factors, as well as biological underpinnings reflected by different genetic, hormonal, and neuronal pathways, were identified (Diehl, 2018; Labaka et al., 2018). Despite the plethora of relevant sex‐related effects in MDD, these differences are not universal and were shown to vary across the lifespan and a wide array of nations (Salk et al., 2017).

Since MDD affects more than 300 million people worldwide representing one of the most frequent medical disorders leading to an immense economic burden, impairments in global functioning and life‐quality (Kraus, Wasserman et al., 2019; Lim et al., 2012; H. U. Wittchen et al., 2011), the present European multicenter study aimed to extend and to specify the current understanding of sex‐related effects in a large naturalistic sample of 1410 patients with MDD as the main diagnosis. The primary objectives were (1) to identify male to female sex ratio in this international sample of MDD patients, and (2) to investigate possible differences in sociodemographic, clinical, and treatment patterns between male and female MDD patients.

## 2 MATERIALS AND METHODS

### 2.1 Design of the study

The present cross‐sectional, observational, noninterventional study with a retrospective assessment of treatment response derives from a large multicenter research consortium named “European Group for the Study of Resistant Depression (GSRD)” (Bartova et al., 2019; Schosser et al., 2012; Souery et al., 1999). The current analyses are based on the GSRD project “Clinical and biological correlates of resistant depression and related phenotypes” performed between 2011 and 2016 across 10 sites (Bartova et al., 2019; Dold et al., 2016) including in‐ as well as outpatient units. Hereby, local ethics committees in Austria, Italy, Belgium, Germany, Greece, France, Israel, and Switzerland approved the study design and all implemented procedures that are comprehensively described in our previous reports (Bartova et al., 2019; Dold et al., 2016). All eligible patients were invited to participate in the present study. The informed consent was signed before enrollment. Since the patient recruitment was conducted in the aforementioned academic centers with an established research focus, that represented a frequent inducement of MDD patients seeking appropriate AD treatment, the number of potentially eligible patients who did not participate in the present study due to their refusal for instance was negligible and was not specifically assessed due to the retrospective, observational, and cross‐sectional study design.

### 2.2 Sample

Adult in‐ and outpatients suffering from a current major depressive episode (MDE) in the course of MDD as the primary diagnosis, established according to the DSM‐IV‐TR (H. Wittchen et al., 1997), were enrolled in the present study. An ongoing adequate psychopharmacotherapy with ≥1 AD agent administered during the current MDE for at least 4 weeks in sufficient daily doses was mandatory for inclusion in the study (Bartova et al., 2019; Dold et al., 2016). MDD patients were excluded when they experienced any other primary psychiatric disorder. The presence of psychiatric and somatic comorbidities was allowed with exception of any severe personality disorder, and/or any substance use disorder present in the previous 6 months.

### 2.3 Collection of data

Sociodemographic, clinical, and treatment characteristics were evaluated by experienced and specifically trained psychiatrists in the course of comprehensive clinical assessments considering also MDD patients' medical records. Hereby, the Mini International Neuropsychiatric Interview (Sheehan et al., 1998) was employed to establish the primary psychiatric diagnosis, psychiatric comorbidities, as well as specific features during the current MDE. Furthermore, the presence of somatic comorbidities was thoroughly assessed with a specific focus on thyroid disease and diabetes due to their close relation to MDD (Fugger et al., 2018, 2019). If applicable, the disease onset, as well as the related treatment, were evaluated.

The severity of depressive symptoms at study entry was evaluated using the Montgomery and Åsberg Depression Rating Scale (MADRS; Montgomery & Asberg, 1979) and the 21‐item Hamilton Rating Scale for Depression (HAM‐D; Hamilton, 1960). To assess the severity of depressive symptoms at the onset of the current MDE, retrospective MADRS (rMADRS) scores based on the MDD patients' assertions and clinical information from their medical records were retrieved.

Treatment response was evaluated based on the MADRS total score change during the current MDE (rMADRS–current MADRS) after at least one adequate AD trial administered ≥4 weeks at sufficient daily doses. The definition of response to AD treatment included a MADRS total score of less than 22 and a ≥50% MADRS total score reduction after an adequate AD trial. While a MADRS total score of ≥22, and a <50% MADRS total score reduction after one adequate AD trial were mandatory for nonresponse, the definition for treatment‐resistant depression (TRD) required a nonresponse to ≥2 consecutive adequate AD trials (Bartova et al., 2019). To warrant a high level of inter‐rater reliability, all psychiatrists involved in the study underwent special training in performing the MADRS.

To evaluate current suicidal risk and, if present its degree, HAM‐D item 3 (suicidality) ratings were employed (Dold, Bartova, Fugger et al., 2018). While the absence of the current suicidal risk was represented by an item‐score of 0 (absent), its presence was reflected by item scores of 1‐4 (1 = feels life is not worth living; 2 = wishes to be dead or any thoughts of possible death to self; 3 = suicide ideas or gestures; 4 = suicide attempts). Subsequently, low suicidality levels were defined by an item score of 1, and moderate to high suicidality levels were characterized by item scores of 2–4.

### 2.4 Statistical Computation

Patients were subdivided into two groups according to their sex (male and female MDD patients). The related sociodemographic, clinical, and treatment characteristics are presented with descriptive statistics (means, *SD*, and/or percentages). Differences between the two sex groups were analyzed using* χ* ^2^ tests for categorical variables and analysis of variance (ANOVA) for continuous variables. The Bonferroni–Holm correction for multiple comparisons was applied. In case of statistical significance, that was set at a *p* ≤ .05, logistic regression analyses (for categorical variables) and analysis of covariance (ANCOVA) for continuous variables considering age and research center as covariates were conducted *post hoc*. The present analyses were performed using version 27 of IBM SPSS Statistics.

## 3 RESULTS

### 3.1 Sample

One thousand four hundred and ten MDD patients were included in the present study, whereby 33.1% (*N* = 467) were male and 66.9% (*N* = 943) were female. The sociodemographic, clinical, and treatment patterns of the whole sample were demonstrated in our previous reports (Bartova et al., 2019) and are displayed in Table 1 split by sex.

**Table 1:** MDD patients' (Bartova et al., 2019) sociodemographic, clinical, and treatment characteristics itemized according to their sex

MDD patients' characteristics	MDD sample in total (*n* = 1410)	Male MDD patients (*n* = 467)	Female MDD patients (*n* = 943)	*χ*^2^/*F*	*p* Value (χ2/ANOVA)	AOR (95% CI)/F	*p* Value (regression analyses/ANCOVA)
Age, mean (*SD*), years	50.3 (14.1)	50.4 (13.8)	50.2 (14.3)	0.0	.890
Ethnic origin, *n* (%)							
Caucasian	1356 (96.2)	451 (96.6)	905 (96)	0.3	.578		
Educational status, *n* (%) (*n* = 1395)							
University education/nonuniversity high education/high‐level general education	755 (53.5)	267 (57.5)	488 (52.4)	3.3	.070
General secondary/technical education/elementary school/none	640 (45.4)	197 (42.5)	443 (47.6)
Occupational status, *n* (%) (*n* = 1408)							
Employed	659 (46.7)	231 (49.5)	428 (45.5)	2.0	.159		
Unemployed	749 (53.1)	236 (50.5)	513 (54.5)				
Relationship status, *n* (%)							
With ongoing relationship	703 (49.9)	252 (54)	451 (47.8)	4.7	.03	0.785 (0.627–0.984)	.036
Without ongoing relationship	707 (50.1)	215 (46)	492 (52.2)				
Major depressive episode, *n* (%)							
Single	127 (9.0)	39 (8.4)	88 (9.3)	0.4	.545		
Recurrent	1283 (91.0)	428 (91.6)	855 (90.7)				
Specific features, *n* (%)							
Psychotic features	154 (10.9)	57 (12.2)	97 (10.3)	1.2	.277
Melancholic features	856 (60.7)	291 (62.3)	565 (59.9)	0.8	.386
Atypical features	33 (2.3)	9 (1.9)	24 (2.5)	0.5	.470
Catatonic features	7 (0.5)	2 (0.4)	5 (0.5)	0.1	.798
Suicidality^a^							
Current suicidal risk (dichotomous)	649 (46.0)	228 (48.8)	421 (44.6)	2.2	.139		
Degree of suicidal risk in patients with current suicidal risk, *n* (%) (*n* = 649)							
High/moderate	377 (58.1)	145 (63.6)	232 (55.1)	4.4	.036	0.694 (0.497–0.970)	.033
Low	272 (41.9)	83 (36.4)	189 (44.9)
Treatment setting, *n* (%)							
Inpatient	488 (34.6)	190 (40.7)	298 (31.6)	11.4	**<.001**	0.713 (0.555–0.917)	**.008**
Outpatient	922 (65.4)	277 (59.3)	645 (68.4)				
Duration of the current MDE, mean (*SD*), days	204.7 (164.6)	200.4 (165.5)	206.9 (164.3)	0.4	.536
Number of MDEs during lifetime, mean (*SD*)	3.3 (2.5)	3.2 (2.5)	3.4 (2.4)	0.7	.412		
Age of MDD onset, mean (*SD*), years	37.2 (15.4)	38.9 (15.4)	36.4 (15.4)	7.6	.006	13.2	<.001
Duration of psychiatric hospitalizations during lifetime, mean (*SD*), weeks (*n* = 1328)	5.6 (20.5)	6.0 (23.9)	5.4 (18.5)	0.2	.641		
Psychiatric comorbidities, *n* (%)							
Any anxiety disorder	294 (20.9)	101 (21.6)	193 (20.5)	0.3	.614
Generalized anxiety disorder	151 (10.7)	51 (10.9)	100 (10.6)	0.0	.857
Panic disorder	114 (8.1)	35 (7.5)	79 (8.4)	0.3	.567
Agoraphobia	113 (8.0)	36 (7.7)	77 (8.2)	0.1	.766
Social phobia	45 (3.2)	17 (3.6)	28 (3.0)	0.5	.500
Obsessive–compulsive disorder	22 (1.6)	6 (1.3)	16 (1.7)	0.3	.565
Posttraumatic stress disorder	20 (1.4)	5 (1.1)	15 (1.6)	0.6	.437
Somatic comorbidities, *n* (%)							
Any somatic comorbidity	653 (46.3)	197 (42.2)	456 (48.4)	4.8	.029	1.336 (1.056–1.690)	.016
Hypertension	267 (18.9)	102 (21.8)	165 (17.5)	3.8	.05		
Thyroid dysfunction	204 (14.5)	31 (6.6)	173 (18.3)	34.6	**<.001**	3.466 (2.305–5.210)	**<.001**
Migraine	156 (11.1)	29 (6.2)	127 (13.5)	16.7	**<.001**	2.425 (1.590–3.699)	**<.001**
Diabetes	84 (6.0)	34 (7.3)	50 (5.3)	2.2	.140		
Heart disease	72 (5.1)	31 (6.6)	41 (4.3)	3.4	.066		
Arthritis	65 (4.6)	19 (4.1)	46 (4.9)	0.5	.495		
Asthma	48 (3.4)	7 (1.5)	41 (4.3)	7.7	**.005**	2.990 (1.328–6.730)	**.008**
Pain	8 (0.6)	2 (0.4)	6 (0.6)	0.2	.625		
Severity of depressive symptoms, mean (*SD*)							
HAM‐D total 21‐item at study entry	19.8 (9.1)	19.9 (8.8)	19.7 (9.2)	0.2	.680
MADRS total at study entry (cMADRS)	24.6 (11.3)	24.5 (11.1)	24.7 (11.4)	0.1	.763		
MADRS total at onset of the current MDE (rMADRS)	34.1 (7.7)	34.5 (7.6)	33.8 (7.7)	2.5	.117
Treatment response, *n* (%)^b^							
Response	346 (24.5)	121 (25.9)	225 (23.9)	2.4	.300		
Nonresponse	492 (34.9)	150 (32.1)	342 (36.3)				
Resistance	572 (40.6)	196 (42.0)	376 (39.9)				
MADRS total change (rMADRS–cMADRS), mean (*SD*)	−9.4 (10.8)	−10.0 (10.7)	−9.0 (10.8)	2.4	.124		
Ongoing psychotherapy, *n* (%) (*n* = 1279)							
Any psychotherapy	399 (28.3)	128 (30)	271 (31.8)	0.4	.505
Cognitive‐behavioral therapy	292 (20.7)	96 (22.5)	196 (23)	4.0	.409
Psychoanalytic psychotherapy	43 (3.0)	16 (3.7)	27 (3.2)
Systemic psychotherapy	16 (1.1)	6 (1.4)	10 (1.2)
Other psychotherapy	48 (3.4)	10 (2.3)	38 (4.5)
Ongoing psychopharmacotherapy							
Number of concurrently administered psychopharmacotherapeutics, mean (*SD*)	2.18 (1.2)	2.3 (1.2)	2.1 (1.2)	3.2	.073		
Administered first‐line antidepressant (in the current MDE), *n* (%)							
Selective serotonin reuptake inhibitors	734 (52.1)	233 (49.9)	501 (53.1)	1.3	.252
Serotonin‐norepinephrine reuptake inhibitors	336 (23.8)	97 (20.8)	239 (25.3)	3.6	.058
Noradrenergic and specific serotonergic antidepressants	121 (8.6)	57 (12.2)	64 (6.8)	11.7	**<.001**	0.550 (0.375–0.807)	**.002**
Tricyclic antidepressants	74 (5.2)	29 (6.2)	45 (4.8)	1.3	.254
Agomelatine	69 (4.9)	18 (3.9)	51 (5.4)	1.6	.203
Noradrenaline‐dopamine reuptake inhibitors	32 (2.3)	15 (3.2)	17 (1.8)	2.8	.094
Serotonin antagonist and reuptake inhibitors	28 (2.0)	10 (2.1)	18 (1.9)	0.1	.768
Vortioxetine	6 (0.4)	3 (0.6)	3 (0.3)	0.8	.379
Monoamine oxidase inhibitors	5 (0.4)	2 (0.4)	3 (0.3)	0.1	.743
Noradrenaline reuptake inhibitors	3 (0.2)	2 (0.4)	1 (0.1)	1.5	.217
Tianeptine	2 (0.1)	1 (0.2)	1 (0.1)	0.3	.612
Fluoxetine equivalents^c^, mean (*SD*), mg/day	39.86 (20.8)	42.6 (23.7)	38.5 (19.0)	11.2	**<.001**	10.0	**.002**
Employed psychopharmacotherapeutic combination and augmentation strategies (in addition to the ongoing antidepressant treatment), *n* (%)							
Any combination and augmentation treatment	855 (60.6)	304 (65.1)	551 (58.4)	5.8	.016	0.771 (0.607–0.979)	.033
Combination with at least 1 additional antidepressant	416 (29.5)	157 (33.6)	259 (27.5)	5.7	.017		
Augmentation with at least 1 antipsychotic drug	362 (25.7)	132 (28.3)	230 (24.4)	2.5	.117		
Augmentation with at least 1 mood stabilizer	159 (11.3)	64 (13.7)	95 (10.1)	4.1	.043	0.685 (0.486–0.966)	.031
Augmentation with pregabalin	102 (7.2)	33 (7.1)	69 (7.3)	0.0	.864		
Augmentation with at least 1 low‐potency antipsychotic^d^	91 (6.5)	25 (5.4)	66 (7.0)	1.4	.237		
Augmentation with benzodiazepines including zolpidem and zopiclone	466 (33.0)	148 (31.7)	318 (33.7)	0.6	.446		

*Note*: The *p* values indicated in bold were significant after Bonferroni–Holm correction.

Abbreviations: ANCOVA, analysis of covariance; ANOVA, analysis of variance; AOR, adjusted odds ratio; CI, confidence interval; HAM‐D, Hamilton Depression Rating Scale; MADRS, Montgomery Åsberg Depression Rating Scale (cMADRS, current MADRS; rMADRS, retrospective MADRS); MDD, major depressive disorder; MDE, major depressive episode; *n*, number of participants; *SD*, standard deviation.

^a^ The presence of the current suicidal risk was measured based on the HAM‐D item 3 (suicidality) ratings (Dold, Bartova, Fugger et al., 2018). While the absence of the current suicidal risk was based on an item score of 0 (absent), the presence of the current suicidal risk was represented by item scores of 1 (feels life is not worth living), 2 (wishes to be dead, or any thoughts of possible death to self), 3 (suicide ideas or gestures), or 4 (suicide attempts).

^b^ Nonresponse was defined by a previous single failed trial and treatment resistance by two or more failed trials (Bartova et al., 2019).

^c^ Fluoxetine dose equivalents were calculated according to Hayasaka et al. (2015).

^d^ Low‐potency antipsychotics comprise the so‐called low‐potency first‐generation antipsychotics and the second‐generation antipsychotic quetiapine less than 100 mg/day (Bartova et al., 2019).

### 3.2 Sociodemographic, clinical, and treatment patterns itemized by sex

The following sociodemographic, clinical, and treatment characteristics differed significantly between the two sex groups (male vs. female MDD patients; shown in Table 1): relationship status (*p*_uncorrected_ = .03), treatment setting (*p*  < .001), the degree of the current suicidal risk (*p*_uncorrected_ = .036), mean age of MDD onset (*p*_uncorrected_ = .006), any somatic comorbidity (*p*_uncorrected_ = .029), comorbid‐ thyroid dysfunction (*p* < .001), migraine (*p* < .001), and asthma (*p* = .005), as well as first‐line AD treatment with noradrenergic and specific serotonergic ADs (NaSSAs; *p*  < .001), mean daily doses of the administered first‐line ADs calculated as fluoxetine dose equivalents (*p*  < .001) according to Hayasaka et al. (2015), any combination and/or augmentation treatment in general (*p*_uncorrected_ = .016), as well as AD combination treatment (*p*_uncorrected_ = .017) and augmentation with mood stabilizers (MS; *p*_uncorrected_ = .043) in particular. All the abovementioned contrasts that withstood the Bonferroni–Holm correction for multiple comparisons remained significant, when age and research center were considered as covariates in our *posthoc* analyses (shown in Table 1), and are described below in more detail.

#### 3.2.1 Sociodemographic and clinical patterns

Male MDD patients were more frequently treated as inpatients (40.7% vs. 31.6%), whereas a higher proportion of outpatients was detected in female MDD patients (68.4% vs. 59.3%). Both sex groups did not significantly differ in terms of the current suicidal risk (*p* = .139), which, however, varied in its degree, whereby moderate to high suicidality levels were revealed in men as compared to women (63.6% vs. 55.1%) who largely exhibited low suicidality levels (44.9% vs. 36.4%). With respect to comorbidities, the co‐occurrence of thyroid dysfunction (18.3% vs. 6.6.%), migraine (13.5% vs. 6.2%), and asthma (4.3% vs. 1.5%) were higher in females as compared to male MDD patients. Focusing on psychopharmacotherapy, mean daily doses of the administered first‐line ADs were higher in males (42.6 ± 23.7) than in females (38.5 ± 19.0) MDD patients. With respect to the first‐line AD treatment, male MDD patients (12.2%) received NaSSAs more frequently as compared to their female counterparts (6.8%). Furthermore, we identified a trend towards a more frequent administration of combination and/or augmentation treatment strategies in men (65.1%) than in women (58.4%). More specifically, AD combination treatment (33.6% vs. 27.5%) and augmentation with MS (13.7% vs. 10.1%) were more often used in male MDD patients.

## 4 DISCUSSION

The present European multicenter study investigated sex‐related effects in 467 male and 943 female patients with MDD as the primary diagnosis. As compared to females, male MDD patients were rather treated as inpatients, suffered from moderate to high suicidality levels, received NaSSAs as first‐line AD treatment, and generally higher mean daily doses of the administered ADs. Furthermore, a trend towards a more frequent administration of add‐on treatments was detected in males. Female MDD patients were rather treated as outpatients and experienced low suicidality levels, comorbid thyroid dysfunction, migraine, and asthma as compared to males. A trend towards earlier mean age of MDD onset was observed in females.

Our results revealed that the majority of MDD patients were females and are underlined by a plethora of previous studies reporting higher occurrence of MDD in women than in men (Kornstein, 1997; Parker & Brotchie, 2010) which were, to some extent, previously explained by potential underdiagnosing of men suffering from depressive symptoms (Moller‐Leimkuhler, 2009). A recent meta‐analysis investigating 3,630,259 individuals reported that sex differences identified in terms of rates and symptoms of MDD vary across the lifespan and a wide array of nations (Salk et al., 2017). Hereby, they were shown to be more profound in nations with greater sex equity (Salk et al., 2017). This might correspond with findings from our multicenter study investigating MDD patients in Austria, Italy, Belgium, Germany, Greece, France, Israel, and Switzerland, representing countries with rather high sex equity, whereby the identified contrasts between men and women largely remained robust, even if research centers were considered as covariates in our *posthoc* analyses. Looking more specifically at the impact of age, the meta‐analysis showed that sex differences peak in adolescence at age 16 and stabilize in adulthood (Salk et al., 2017). This is in line with our study revealing no contrasts in terms of symptom severity assessed with MADRS and HAM‐D, treatment response, disease chronicity reflected by the number of previous MDEs and the duration of psychiatric hospitalizations, as well as psychiatric comorbidities, and specific features during the current MDE between male and female MDD patients, who were at a mean age of 50.3 ± 14.1 and who experienced their first MDE at a mean age of 37.2 ± 15.4, representing an adult age of disease onset. In line with previous evidence (Azorin et al., 2014; Fava et al., 1993), women experienced their first MDE at a younger age than men, whereby the identified contrast gained in significance, when age and research center were considered as covariates.

In accordance with our findings, a previous German survey focusing on male depressive symptoms in a largely comparable sample of 2411 adult MDD patients reported no sex differences with respect to the duration and severity of MDD as well as its first hospitalization which, however, emerged when symptom patterns were considered (Moller‐Leimkuhler et al., 2004). This seems to be of importance since there is further evidence showing that comorbid anxiety and atypical features occurred more frequently in female MDD patients (Grigoriadis & Robinson, 2007; Marcus et al., 2005). The fact that these studies, including the Sequenced Treatment Alternatives to Relieve Depression (STAR*D) study for instance (Marcus et al., 2005), largely investigated MDD patients with rather a chronic disease course may explain the difference to our MDD patients who were not chronically ill and who did not show sex‐related effects in terms of psychiatric comorbidities and specific phenomena as melancholic, psychotic, atypical, and catatonic features. As compared to previous literature, lacking contrasts in such relevant clinical conditions in our study might be additionally elucidated by increasing equity in important psychosocial factors and roles that were attributed to either men or women for years and that were, accordingly, thought to lead to typical male and female clinical manifestation of MDD (Moller‐Leimkuhler, 2009; Moller‐Leimkuhler & Yucel, 2010).

However, female MDD patients included in our study showed higher rates of somatic comorbidities including thyroid dysfunction, migraine, and asthma which may, in turn, correspond with the STAR*D study associations between female MDD patients and somatoform disorders (Marcus et al., 2005). Importantly, further evidence reported a higher occurrence of cardiovascular diseases (CVDs) in female MDD patients, which was ascribed to greater exposure to chronic stressors, internalizing coping styles, and interpersonal stress responsiveness in women (Moller‐Leimkuhler, 2010). Looking at CVD more specifically, sex‐related effects, however, seem not to be that conclusive and universal and hence, should be interpreted under consideration of further important clinical, biological, socioeconomic, and psychosocial factors as the primary diagnosis, comorbidities, coping strategies, and help‐seeking behavior for instance (Moller‐Leimkuhler, 2007, 2008). In this context, we are also aware of further reports observing higher rates of comorbid CVD in male MDD patients (Azorin et al., 2014) which, in turn, resemble our nonsignificant findings revealing a higher proportion of male MDD patients suffering from comorbid hypertension and heart disease in general. Considering other perspectives, CVD was previously associated with the so‐called “type A personality” characterized by specific traits including increased impulsivity (Abbott et al., 1984) that was repeatedly linked to male sex in MDD (Azorin et al., 2014; Oliffe et al., 2019).

Consistently, higher impulsivity and related clinical phenomena as anger attacks (Winkler et al., 2004; 2005) and suicidality with a higher number of completed suicides (Rutz et al., 1995) were repeatedly observed in men suffering from MDD and hence, were suggested as core symptoms of the so‐called male depression (Moller‐Leimkuhler, 2009), requiring special attention especially in the course of preventive and therapeutic approaches (Oliffe et al., 2019; Rihmer et al., 1995). This seems to be of particular importance when the paradox of low rates for MDD and, concurrently, high rates for completed suicides in men is considered (Moller‐Leimkuhler, 2003, 2009; Rutz et al., 1995). The results of the present study revealing a lower proportion of men in the whole sample of MDD patients on the one hand, and a moderate to a high degree of the current suicidal risk in male MDD patients on the other hand, may underline the concept of male depression or at least divergent sex‐related patterns in terms of suicidality in MDD.

Considering psychopharmacotherapy, our male MDD patients more frequently received NaSSAs as their first‐line AD treatment, and further add‐on strategies as AD combination treatment and augmentation with MS which, however, did not withstand the correction for multiple comparisons. Importantly, administering NaSSAs as ADs and MS as augmenting agents was repeatedly shown to exert potent AD effects and to be very effective in terms of impulsivity and suicidality (Bauer et al., 2017; Fava et al., 1993), which might be further potentiated in the course of AD combination treatment (Dold, Bartova, Fugger et al., 2018; Dold, Bartova, Kautzky et al., 2018; Dold, Bartova, Mendlewicz et al., 2018; Dold & Kasper, 2017). Since our male and female MDD patients differed in terms of low and moderate/high suicidality levels, but the current suicidal risk *per se* was comparable between both sex groups, the prescription of the aforementioned treatment options in our depressed men might reflect an effective use of recommended psychopharmacotherapeutic strategies to prevent this serious medical condition. Alternatively, clinicians may have followed the suggested specificity of AD effects of selective serotonin reuptake inhibitors (SSRIs) in females (Sramek et al. 2016). Furthermore, patients' preferences should be taken into account since they play a major role in adherence to the prescribed AD treatment (Dold & Kasper, 2017). Hypothetically, the less frequent prescription of NaSSAs in female MDD patients could be associated with their general cautious attitude against substances which may cause an increase in appetite (Schneier et al., 2015) or even weight gain. Focusing on the particular augmenting agents, it is worthwhile to look at their pharmacological properties as well as the general prescription recommendations. Theoretically, the less common administration of MS in female MDD patients, representing 67% of our sample, could be related to the fact that this substance class was reflected by valproic acid (VPA) in a substantial number of cases (Dold et al., 2016). VPA, however, is contradicted in women of childbearing age due to its potential teratogenic effects (Gentile, 2010).

Importantly, the identified robust contrasts between both sexes including treatment setting, first‐line AD treatment with NaSSAs, daily doses of the administered first‐line ADs, and comorbid thyroid dysfunction, migraine, and asthma seem to be crucial, since clinical patterns as being an inpatient, suffering from a higher number of comorbidities, first‐line AD treatment with agents other than SSRIs, and the necessity of higher dosing were repeatedly related to TRD and the so‐called difficult‐to‐treat depression (Bartova et al., 2019; Kraus, Kadriu, et al., 2019; McAllister‐Williams et al., 2020). In our study, these rather unfavorable disease characteristics were largely observed in men, even though they did not differ from women in terms of treatment response. However, the fact that inpatient treatment and higher daily doses of the first‐line ADs were more frequently required in our male MDD patients may reflect increased therapeutic efforts, which were undertaken to avoid nonresponse or even TRD, though these variables did not significantly differ between men and women in our study. With respect to AD daily doses, it is noteworthy that high‐dose treatment, that is also called “dose escalation,” did not show superiority over the continuation of standard‐dose treatment in MDD (Dold et al., 2017) and hence, does not represent evidence‐based treatment for TRD (Bauer et al., 2017; Dold & Kasper, 2017) despite its common utilization in the clinical routine (Dold et al., 2016).

Focusing on the potential strengths and limitations of the present report, the international, multicenter, and cross‐sectional study design has to be prioritized. Our sample represents a real‐world population of MDD patients experiencing a wide range of disease course, symptom severity, and clinical manifestations including suicidality and comorbidities (Bartova et al., 2019). Especially the presence of the latter clinical phenomena differentiates the present study from the majority of randomized controlled trials applying rather stringent inclusion criteria that did not allow such challenging clinical heterogeneity. In spite of the real‐world MDD patient characteristics, our sample may not be fully representative for depressed patients seeking help in primary care, since the recruitment was conducted in academic centers with an established research focus, and since an adequate AD treatment employed for at least 4 weeks was mandatory for enrollment in the present study that predominantly focused on TRD. As data on potentially eligible patients who did not participate in the study is not available, a minor selection bias cannot fully be ruled out. Further limitations refer to the evaluation of treatment response that exclusively considered psychopharmacotherapy and that included rMADRS scores, which are not as precise as prospective assessments. Additionally, it should be taken into account that the present study focused on the investigation of sociodemographic and clinical parameters, while biological factors as genetic, hormonal, or neuronal pathways, which were shown to play an important role in the explanation of sex‐related effects in MDD, were not considered.

## 5 CONCLUSION

The aforementioned benefits, as well as limiting aspects of the present study, should be critically taken into account when the complex interaction of sex with the clinical phenotype of MDD is interpreted. Being aware that the present study was not specifically designed to examine sex‐related effects in MDD, we identified moderate to high suicidality levels and the necessity of greater therapeutic efforts in terms of inpatient treatment as well as more complex psychopharmacotherapy in men, and higher rates of somatic comorbidities in women. Such subtle clinical divergencies may contribute to the concept of male and female depressive syndromes and may further serve as predictors of disease severity and course, as they represent core symptoms of MDD or phenomena that were repeatedly related to TRD. Most importantly, considering sex may guide AD treatment towards targeting specific clinical manifestations including comorbidities, as well as management of challenging conditions as suicidality, and importantly, prevention of treatment resistance and chronicity.

## CONFLICT OF INTERESTS

Dr. Bartova has received travel grants and consultant/speaker honoraria from AOP Orphan, Medizin Medien Austria, Vertretungsnetz, Schwabe Austria, Janssen and Angelini. Dr. Dold has received travel grants and consultant/speaker honoraria from Janssen‐Cilag. Dr. Frey has received consulting fees from Janssen‐Cilag. Dr. Zohar has received grant/research support from Lundbeck, Servier, and Pfizer; he has served as a consultant or on the advisory boards for Servier, Pfizer, Solvay, and Actelion; and he has served on speakers' bureaus for Lundbeck, GlaxoSmithKline, Jazz, and Solvay. Dr. Mendlewicz is a member of the board of the Lundbeck International Neuroscience Foundation and of the advisory board of Servier. Dr. Souery has received grant/research support from GlaxoSmithKline and Lundbeck; and he has served as a consultant or on advisory boards for AstraZeneca, Bristol‐Myers Squibb, Eli Lilly, Janssen, and Lundbeck. Dr. Montgomery has served as a consultant or on advisory boards for AstraZeneca, Bionevia, Bristol‐Myers Squibb, Forest, GlaxoSmithKline, Grunenthal, Intellect Pharma, Johnson and Johnson, Lilly, Lundbeck, Merck, Merz, M's Science, Neurim, Otsuka, Pierre Fabre, Pfizer, Pharmaneuroboost, Richter, Roche, Sanofi, Sepracor, Servier, Shire, Synosis, Takeda, Theracos, Targacept, Transcept, UBC, Xytis, and Wyeth. Dr. Fabbri has been supported by Fondazione Umberto Veronesi (https://www.fondazioneveronesi.it). Dr. Serretti has served as a consultant or speaker for Abbott, Abbvie, Angelini, AstraZeneca, Clinical Data, Boehringer, Bristol‐Myers Squibb, Eli Lilly, GlaxoSmithKline, Innovapharma, Italfarmaco, Janssen, Lundbeck, Naurex, Pfizer, Polifarma, Sanofi, and Servier. Within the last 3 years, Dr. Kasper received grants/research support, consulting fees, and/or honoraria from Angelini, Celegne GmbH, Eli Lilly, Janssen‐Cilag Pharma GmbH, KRKA‐Pharma, Lundbeck A/S, Mundipharma, Neuraxpharm, Pfizer, Sanofi, Schwabe, Servier, Shire, Sumitomo Dainippon Pharma Co. Ltd., Sun Pharma, and Takeda. All other authors declare that there are no conflicts of interest.

## AUTHOR CONTRIBUTIONS

Dr. Bartova contributed to designing the study, implementation of the research, statistical analyses, and writing the report including the first draft of the manuscript. Dr. Dold and Dr. Kasper contributed to designing the study, implementation of the research, and writing the report. All authors contributed to designing the study, implementation of the research, and have critically revised and approved the final manuscript.

## Data Availability

The data that support the findings of this study are available from the corresponding author upon reasonable request.
